# The high expression instead of mutation of p53 is predictive of overall survival in patients with esophageal squamous‐cell carcinoma: a meta‐analysis

**DOI:** 10.1002/cam4.945

**Published:** 2016-11-23

**Authors:** Ziran Zhao, Pan Wang, Yibo Gao, Jie He

**Affiliations:** ^1^ National Cancer Center/Cancer HospitalChinese Academy of Medical Sciences and Peking Union Medical CollegeBeijing100021China

**Keywords:** Esophageal squamous‐cell carcinoma, expression, meta‐analysis, overall survival, p53

## Abstract

Esophageal squamous‐cell carcinoma (ESCC) is one of the deadliest cancers where biomarkers are needed for assist guiding management. We performed a meta‐analysis to clarify the prognostic value of p53 high expression and *TP53* mutations, which remain controversial for decades in patients with ESCC. We searched PubMed, Ovid MEDLINE, Embase, and Current Contents Connect to identify studies published between January 1990 and February 2016 of esophageal cancer populations that measured p53 expression and/or mutation status and reported hazard ratios (HRs), or adequate data for estimation of HRs for survival for p53‐defined subgroups. We calculated pooled HR and 95% confidence interval (CI) using a random‐effects model. A total of 56 eligible studies including 6537 patients were identified. The p53 high expression was associated with reduced survival (HR: 1.35, 95% CI: 1.21–1.50, *I* ^2^ = 42%). In subgroup analyses, a greater prognostic effect was observed in those studies that reported survival for pure ESCC cohorts and were assessed at low risk of bias (HR: 1.46, 95% CI: 1.29–1.65, *I* ^2^ = 8%). Patients with ESCC and p53 high expression have reduced overall survival, and this effect is independent of tumor stage and greater than that of *TP53* mutations.

## Introduction

Esophageal cancer is the sixth leading cause of cancer death and the eighth most frequently diagnosed cancer worldwide, and esophageal squamous‐cell carcinoma (ESCC) accounts for about 90% of cases of esophageal cancer worldwide, with a dismal 5‐year survival rates at less than 15% 1. Surgery, combined with neoadjuvant radiation and/or chemotherapy remains the only curative modality for ESCC 2. But fewer than half of the ESCC patients are eligible for this. Even for those patients received curative treatment, 5‐year survival rates are only about 45% 3. Unlike other malignancies, such as lung cancer, where molecular information has become routine practice for therapeutic stratification, current treatment algorithms for ESCC still depend on only imaging and histological assessments 4.

The p53 protein, encoded by the quintessential tumor‐suppressor gene tumor protein 53 (*TP53*) widely regarded as “the guardian of the genome” 5, is one of the most frequently studied proteins in human cancers 6. Substantial efforts have been made to study the effect of p53 expression and/or *TP53* mutation status on prognosis for patients with cancer, but the results remain controversial for decades in patients with ESCC 7, 8. Recently, we and colleagues reported large‐scale genomic sequencing studies showing that ESCC harbors a very high *TP53* mutation rate of up to 90% 9, 10, 11, 12, 13, 14, 15. These mutations, mostly causing p53 single amino acid substitutions, result in expression of full‐length p53 protein, but loss of wild‐type tumor‐suppressive function, indicating a central role of p53 in ESCC. Meanwhile, in esophageal adenocarcinoma (EAC), the second major subtype of esophageal cancer, the prognostic value of *TP53* mutations was clarified better than that of p53 high expression. Hence, to investigate the prognostic value of p53 in ESCC, we conducted a systematic review and meta‐analysis of all publicly available data with subgroup analysis of studies assessed as low risk of bias, and studies using immunohistochemistry (IHC) to determine p53 expression status or sequencing determine *TP53* mutation status.

## Methods

### Literature search and selection of studies

A systematic search to identify eligible studies up to 9 February 2016 was conducted through four databases (PubMed, Ovid MEDLINE, Embase, and Current Contents Connect). The results were integrated with conference abstracts and proceedings retrieved through Web of Science, Embase, Scopus, American Society for Clinical Oncology, American Association for Cancer Research and Digestive Disease Week till 2016. The search strategy included MeSH terms and text words for o/esophagus* or o/esophageal*, p53* or TP53* or 17p* or 17p13*, carcinoma*.

Eligibility criteria for inclusion were the studies evaluated the correlation between p53 expression and/or *TP53* mutation status and overall survival among ESCC patients with calculation of hazard ratios (HR) and 95% confidence interval (CI), or reported sufficient information for their estimation. We also included studies of esophageal cancer cohorts that included squamous‐cell carcinoma patients if more than a half of the patient cohort had a diagnosis of ESCC.

Study exclusion criteria were studies of autoantibody detection in blood or *TP53* DNA germline mutations, and reports available in abstract form only that did not report adequate information to determine study eligibility or to assess study methods for risk of bias.

When multiple studies reported identical or overlapping patient cohorts, only the most recent publication with the largest patient numbers was included in the analysis. When additional information was needed to calculate HRs or the data were missing/unclear, the corresponding author was contacted by email to request for this information.

Two reviewers (Z. Zhao and P. Wang) screened the search results independently and bibliographies of studies were checked for additional relevant articles that may not have been identified by the strategy outlined above.

### Data extraction

Two investigators (Z. Zhao and P. Wang) independently summarized the eligible studies and performed data extraction using a predefined form. The data were composed of study design, study population characteristics, specimen type, tumor type, treatment details, method(s) of p53 status detection, cut‐point or criteria used to define p53 expression status, prevalence of p53 high expression and patient survival outcomes. Disagreements were resolved by discussion. To facilitate further quantitative analyses, the authors’ definitions for p53 “high expression” were used for studies performing only gene sequencing or single‐strand conformation polymorphism (SSCP) as the respective studies did not use uniform assay for mutation detection. Therefore, *TP53* gene mutations were interpreted to represent nuclear p53 protein overexpression by all authors of the included studies, although loss of p53 protein expression has also been associated with *TP53* gene mutations 16. This expression pattern was not reported and/or interpreted in such a manner in any of the included studies.

### Risk of bias assessment

Risk of bias for individual studies was assessed with the risk of bias table recommended by the Cochrane Collaboration 17, which was customized by the criteria proposed by the Grading of Recommendations, Assessment, Development and Evaluation (GRADE) Working Group (www.gradeworkinggroup.org) 18 and REporting recommendations for tumor MARKer prognostic studies (REMARK) 19 to the evaluation of observational studies. And six domains (eligibility criteria, measurement of exposure and outcome, confounding measurement and account, follow‐up, selective outcome reporting, analysis method) were ultimately included in the risk of bias table.

The question for each domain is answered with “Yes” (indicating low risk of bias), “No” (indicating high risk of bias), and “Unclear” (indicating unclear or unknown risk of bias). The overall risk of bias for the study was assessed as high if one or more of the domains was assessed as high risk of bias as recommended by the Cochrane Collaboration. Studies that did not adjust for tumor stage to assess the independent impact of p53 expression status on patient survival were classified as high risk of bias. Details of assessment of the risk of bias for different methods of assessing p53 expression status are summarized in Supporting Information.

### Statistical methods

To statistically evaluate the prognostic effect of p53 expression status on ESCC survival, we pooled the extracted HRs and 95% CIs using the generic inverse variance method. If the HRs and their associated standard errors, CIs, or *P* values were not directly provided in the original articles, we estimated HRs from the corresponding Kaplan–Meier curves using the Parmar method and the statistical data provided in the paper 20, 21. Because we expected interstudy heterogeneity, random‐effects model to estimate the HR 22. Heterogeneity was tested by both *I* ^*2*^ test and Q‐test. The *I* ^2^ more than 50% or Q‐test reporting a *P* value <.1 were defined as heterogeneous 23. We inspect possible sources of interstudy heterogeneity using meta‐regression 24. Study level factors that could modify the prognostic effect of p53 were included as covariates if they were present in ≥30 of the included studies. In the subgroups analyses, which were performed for tumor histology, p53 assay method, and the risk of bias, we repeated the pooled HR analysis to assess their impact on survival. We defined the cut‐point 0%, 5%, and 10% as the “lower cut‐off value”, 20% and higher as the “higher cut‐off value”. To test for differences between subgroups, we performed tests of interaction. Funnel plot analyses were used to evaluate publication bias.

Meta‐analyses of HR estimates were performed using RevMan 5.3 (The Nordic Cochrane Centre, The Cochrane Collaboration, 2014) with meta‐regression performed using the SPSS V.22(IBM Corporation, Somers, NY).

## Results

### Baseline study characteristics

**Figure 1:**
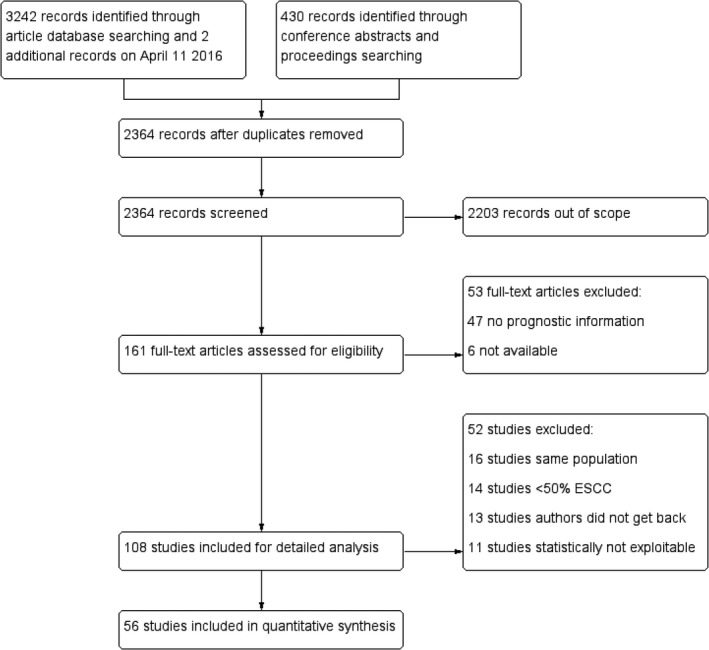
Flowchart summarizing study identification and selection.

According to the literature search and study selection criteria, we identified a total of 3242 studies, of which 56 met our eligibility criteria, comprising 6537 patients for meta‐analysis (Fig. 1). The baseline characteristics of the included studies are summarized in Table S1. The studies included were published between 1993 and 2016. Forty‐seven studies included pure ESCC cohorts while the other nine studies included mixed histological cohorts, in which the percentage of ESCC cases in the study data ranged from 62% to 99%. The sample size of the included studies ranged from 33 to 830 patients (median sample size, 81 patients). Of the total 6537 patients, 6472 were ESCC, with overall survival data reported for 5944 patients. The number of patients with survival data in each study ranged from 33 to 775 (median 79).

The p53 status was assessed by IHC in 45 studies, by next‐generation sequencing (NGS) in five studies, and by *TP53* gene sequencing in four studies. The remaining two studies performed SSCP to assess p53 status. Using these assessing methods, a median of 53% (range 23–96%) of all tumors were classified as p53 high expression. The clinicopathological variables and survival times reported in the included studies are summarized in Table S3. In the 10 studies that reported survival time data for biomarker‐defined patient subgroups, the median survival time for patients assessed as having p53 high expression was 27.9 months.

HRs were reported in 26 studies and extrapolated from 25 studies. In addition, individual patient data were available for five studies (491 patients), and chi‐square and *P* value were available for one study to calculate HR and 95% CIs (Table S1).

Stratified by risk of bias of the included studies, there were 16 studies with a low risk of bias and 40 studies with a high risk of bias (Table S2). Funnel plot analyses were carried out for the analyses of all studies that did not indicate relevant publication bias (Fig. S2).

### Overall analyses

**Figure 2:**
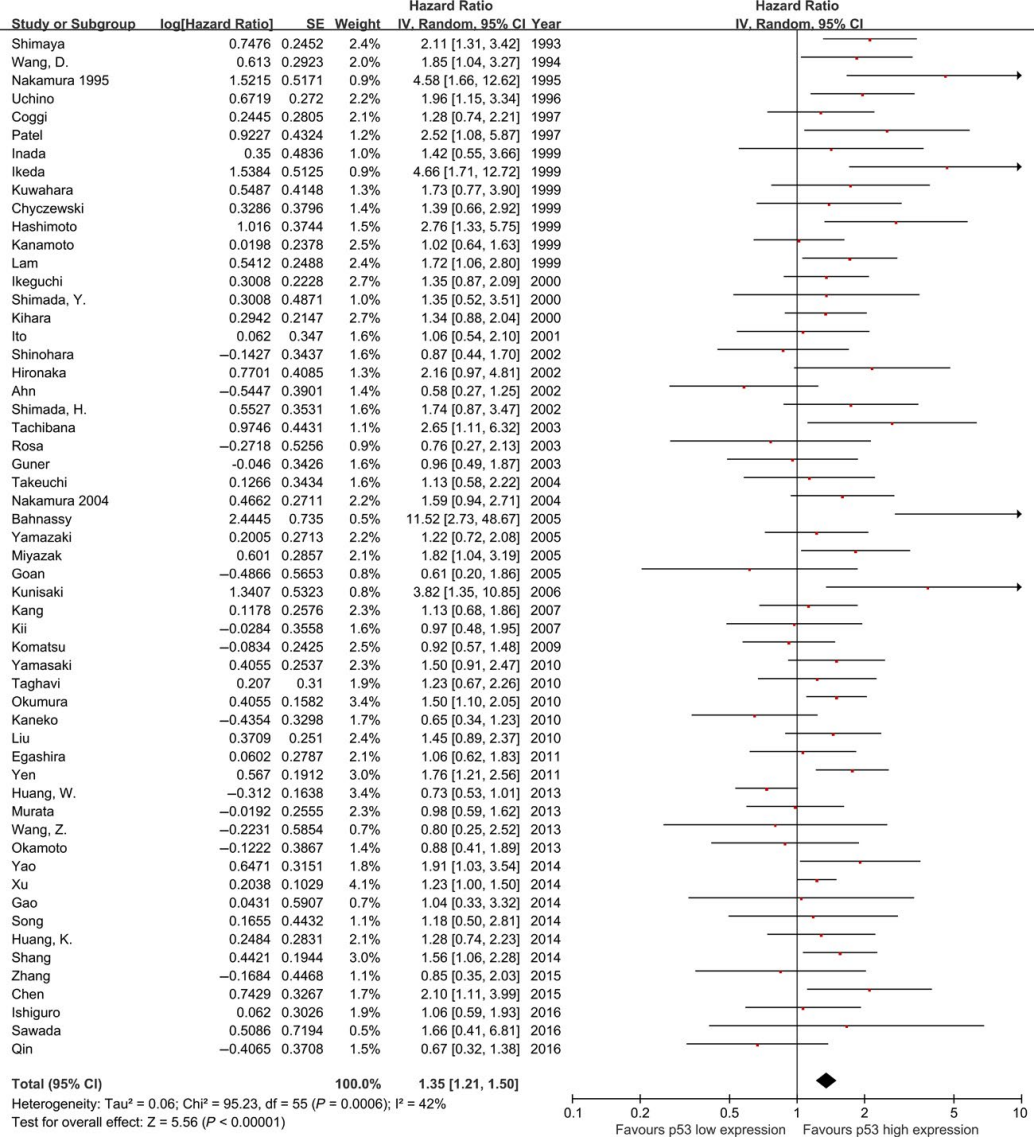
Meta‐analysis of the pooled effect of p53 high expression on survival, all 56 included studies

**Figure 3:**
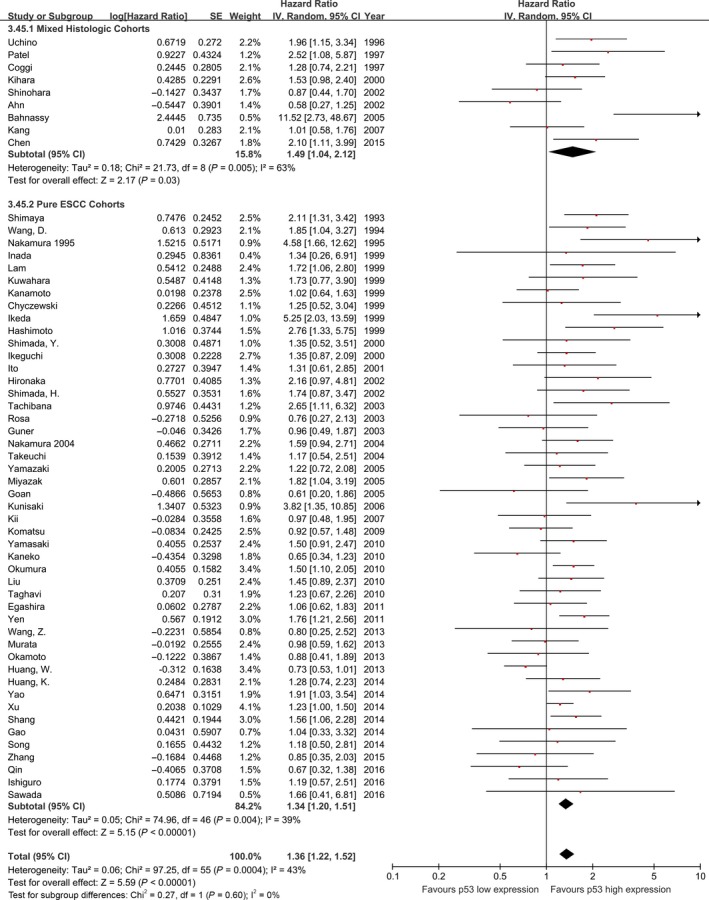
Meta‐analysis of the pooled effect of p53 high expression on survival stratified by tumor histology included in studies.

The meta‐analysis of all 56 studies on overall survival showed a prognostic effect for p53 high expression with an HR 1.35 (95% CI: 1.21–1.50, *I* ^2^ = 42%; Fig. 2), with low‐moderate heterogeneity across studies. Similarly, pooled analysis of studies including pure ESCC cohorts showed p53 high expression is associated with a statistically significant poor outcome with lower heterogeneity (HR: 1.34, 95% CI: 1.20–1.51, *n* = 47, *I* ^2^=39%, *P* for interaction = 0.60; Fig. 3).

### Subgroup and sensitivity analyses

**Figure 4:**
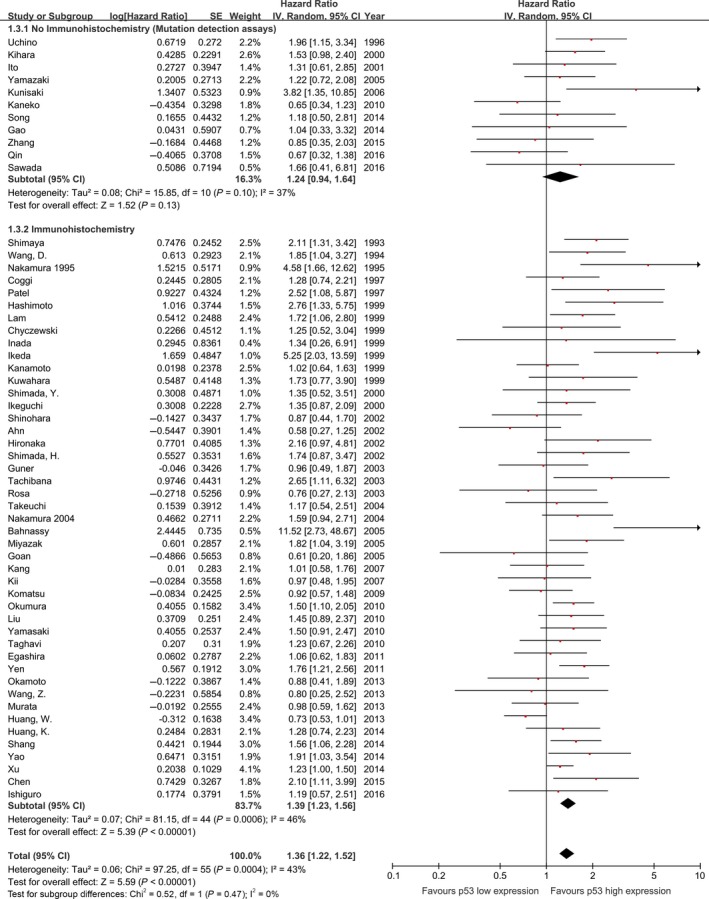
Meta‐analysis of the pooled effect of p53 high expression on survival stratified by p53 expression analysis methodology, including all studies. *The p53 status detected both by IHC and sequencing in these five studies. IHC, immunohistochemistry.

**Figure 5:**
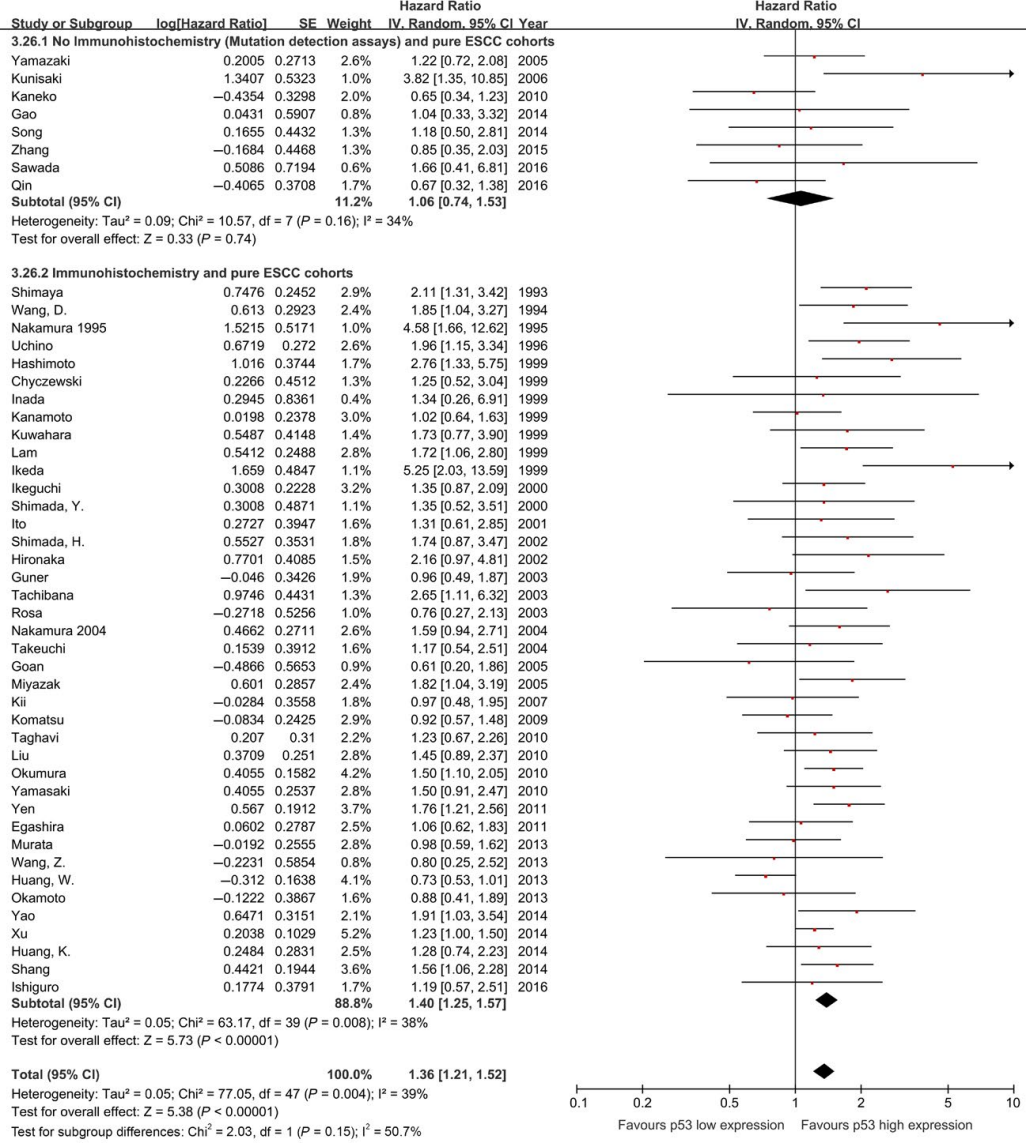
Meta‐analysis of the pooled effect of p53 high expression on survival stratified by p53 expression analysis methodology, only including studies with pure ESCC cohorts. ESCC, esophageal squamous‐cell carcinoma

The effect of p53 status on overall survival appeared to be smaller among studies performing *TP53* gene mutation assessments (sequencing and SSCP) (HR: 1.24, 95% CI: 0.94–1.64, *P* = 0.13, *n* = 11, *I* ^2^ = 37%) compared with studies performing p53 expression assessments with IHC (pooled HR: 1.39, 95% CI: 1.23–1.56, *P* < 0.00001, *n* = 45, *I* ^2^ = 46%; Fig. 4). This finding was similar in studies including pure ESCC cohorts (Fig. 5).

The effect of p53 high expression on patient overall survival was larger in studies that had adjusted their analyses for tumor stage (HR: 1.45, 95% CI: 1.26–1.68, *P* < 0.00001, *n* = 21, *I* ^2^=31%; Fig. S3) compared with the estimates from studies that reported unadjusted risk estimates (HR: 1.28, 95% CI: 1.11–1.49, *P* = 0.0009, *n* = 35, *I* ^2^=46%). A similar effect was seen in the subset of studies containing pure ESCC cohorts (Fig. S3).

**Figure 6:**
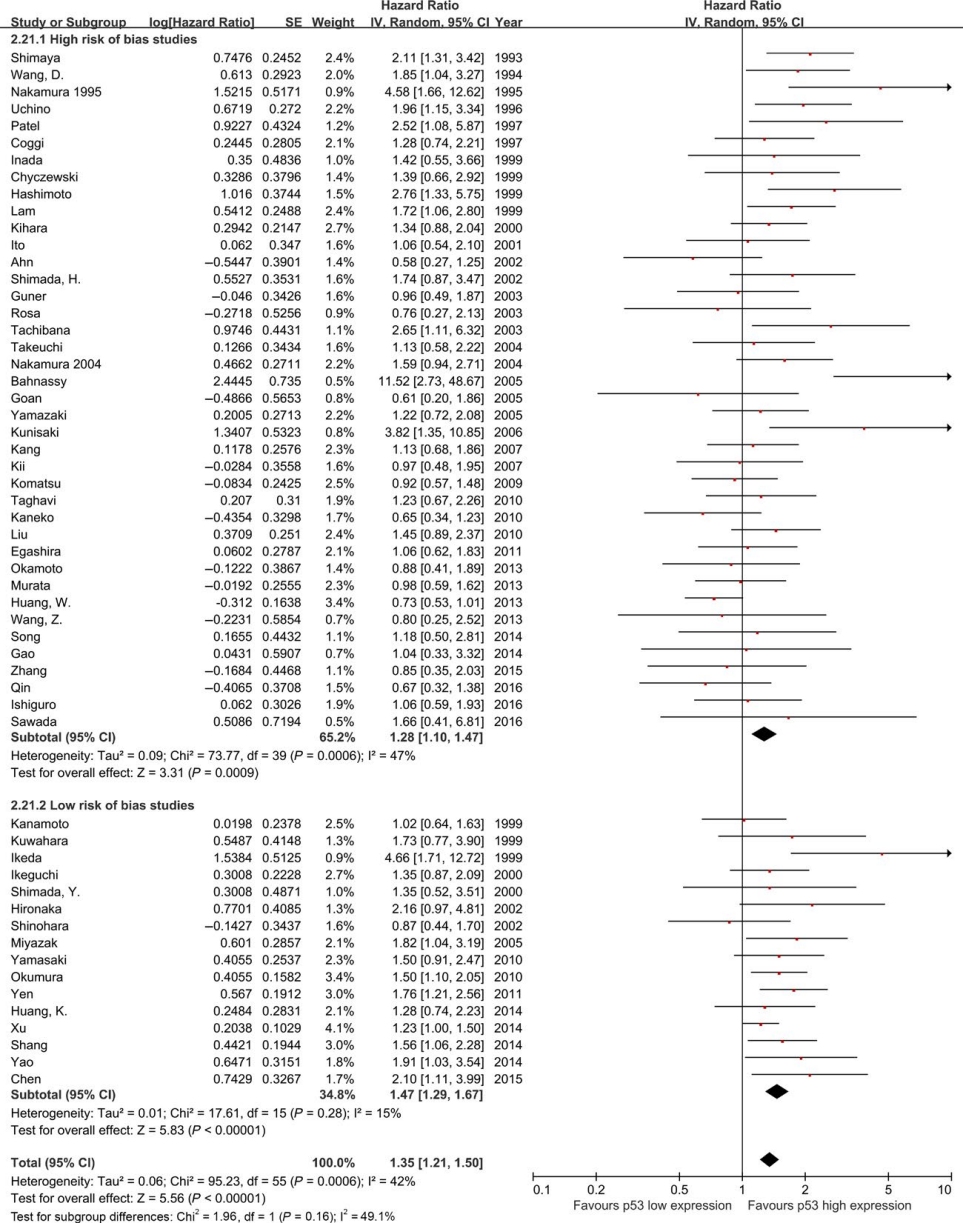
Meta‐analysis of the pooled effect of p53 high expression on survival stratified by risk of bias assessment including, all studies.

**Figure 7:**
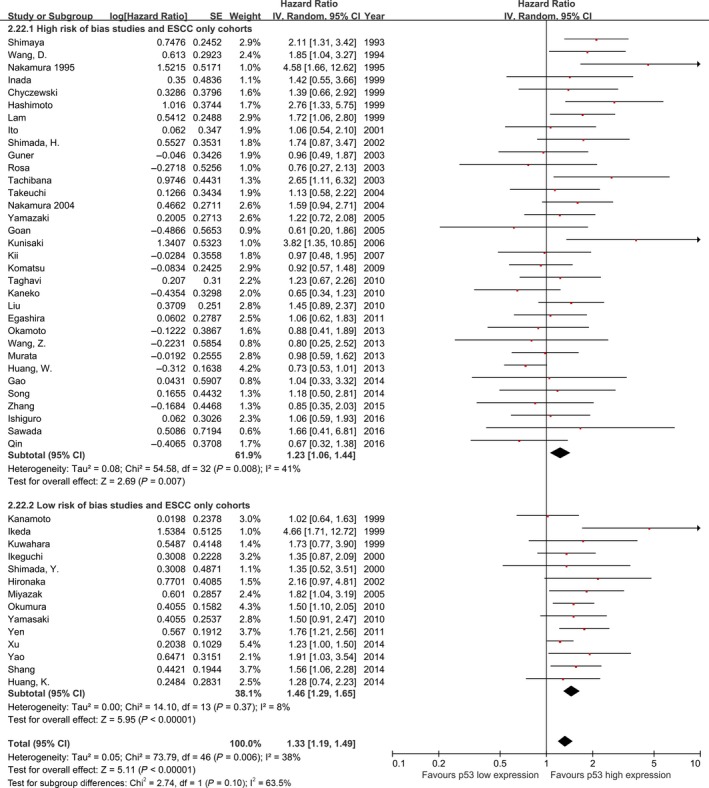
Meta‐analysis of the pooled effect of p53 high expression on survival stratified by risk of bias assessment including, only studies with pure ESCC cohorts. ESCC, esophageal squamous‐cell carcinoma.

The prognostic effect of p53 high expression was more pronounced in the subset of 16 studies assessed as low risk of bias (HR: 1.47, 95% CI: 1.29–1.67, *P* < 0.00001, *I* ^2^ = 15%; Fig. 6), compared with those assessed as high risk of bias (HR: 1.28, 95% CI: 1.10–1.47, *P* = 0.0006, *I* ^2^ = 47%, *P* for interaction = 0.16). This effect size was similar in the 14 studies with low risk of bias that contained pure ESCC cohorts (HR: 1.46, 95% CI: 1.29–1.65, *P* < 0.00001, *I* ^2^ = 8%; Fig. 7).

Subgroup analysis of studies that determined p53 high expression status using IHC by different cut‐off value showed that the prognostic effect of p53 high expression was smaller in the subset of 13 studies with higher cut‐off value (HR: 1.21, 95% CI: 0.99–1.47, *P* = 0.06, *I* ^2^=46%) compared with the estimates from 32 studies with lower cut‐off value. Findings were consistent when the same subgroup analysis was performed for only those p53 IHC studies with lower cut‐off value including pure ESCC cohorts (HR: 1.47, 95% CI: 1.29–1.69; *P* < 0.00001, *n* = 28, *I* ^2^=28%; Fig. S4).

A summary of the results of therapy subgroup analyses can be found in Table S4. Of the 14 inspected study covariates, none were the significant sources of heterogeneity (Table S5).

## Discussion

The present meta‐analysis provides coherent evidence that the high expression instead of mutation of p53 is a significant negative independent prognostic marker in ESCC patients. The pooled HR of 1.35 (95% CI: 1.21–1.50) showed that patients with p53 high expression were expected to have shorter OS. Subgroup analyses revealed that poorer OS (HR: 1.47, 95% CI: 1.29–1.67) with pure ESCC cohorts and low risk of bias.

This is the first and most full‐scale meta‐analysis systemically exploring the independent prognostic role of p53 high expression in patients with ESCC. Similar significant negative effect estimates were reported in two previous meta‐analyses, but potential confounders such as tumor stage were not considered and the studies and patients included were relative less 25, 26. Thus, our meta‐analysis, with 56 studies with records of more than 6500 patients, is the most full‐scale and extensive analysis of the effect of p53 high expression on ESCC patient survival.

Recently, NGS studies have shown a high mutation rate of *TP53* in ESCC, as high as 93% in our cohort 10. *TP53* mutation rate increased with usage of this advanced sequencing assay (Fig. S7). The prognostic value of *TP53* mutation has been proved in various types of cancer 27. There might be poorer prognosis in patients with *TP53* mutation. Thus, the high *TP53* mutation rate could be one potential explanation for the poor prognosis of ESCC patients. However, *TP53* mutation is so common that we cannot stratify ESCC patients based on *TP53* mutation alone. Differential survival outcomes were observed in patients with different types of *TP53* mutation 28, such as mutation location in ovarian and breast cancers 29. In the subgroup of ESCC NGS studies, we compared the over survival of patients with different *TP53* mutation number (0 vs. 1 vs. >1), spectrum, and allele frequency (<50% vs. >50% etc.). But there were no differential survival outcomes in these analysis, as well as in different p53 domains (data not shown). However, this meta‐analysis results showed p53 expression status were stronger prognostic significance than *TP53* mutation in patients with ESCC. Similar results were found in the p53 status detection methods subgroup analysis including the studies detecting both expression and mutation (Fig. S5 and S6). We could stratify ESCC patients based on p53 expression status instead of mutation.

For ESCC patients, p53 expression status is not only predictive of overall survival, but also might be clinically relevant in therapeutic regimens selection. It has been proved that p53 expression status could predict response to chemotherapy regimens such as cisplatin and 5‐fluorouracil, both in ESCC and other cancer type patients 7, 8, 30. A variety of p53‐directed therapeutic approaches for esophageal and other cancer patients, such as APR‐246, are currently in clinical trials. Some of them are even ready to be available in clinical therapy 31. As the high frequency of *TP53* mutation in ESCC, and evidence from recent studies showed that p53 missense mutations abrogate its tumor‐suppressive function and lead to a “gain‐of‐function” that promotes cancer, it might be worthwhile to try those approaches in the treatment of this deadly cancer 6, 32, 33.

The strengths of this meta‐analysis relate to the comprehensive search and rigorous approach that included selecting and appraising studies by an independent pair of reviewers. However, certain limitations may affect the validity of our findings. First, we were unable to explore the effect of other potentially relevant factors (such as patient smoking and drinking status) because of the lack of data. Furthermore, IHC methods in the included studies were variable, such as different kind of antibodies, dilutions, and cut‐off values for p53 high expression. But in all pooled and subgroup analyses, the key finding of p53 prognostic value still exist. Last, potential publication bias might exist despite conducting an extensive search strategy and presenting a funnel plot that excludes major asymmetry.

ESCC is still one of the deadliest cancers which biomarkers are needed for assist guiding management. The present meta‐analysis shows that p53 high expression instead of mutation is predictive of overall survival in ESCC patients. In future, more well‐designed studies with large patient cohorts using more precise technologies for p53 expression status detection are still required to verify the conclusion.

## Conflict of Interest

None declared.

## Supporting information


**Figure S1**. Analysis of ESCC patients in the IARC TP53 mutation database. Figure S1A depicts the corresponding IHC staining patterns of the type of TP53 gene mutations. Figure S1B shows how the TP53 mutation effect affects IHC staining patterns, where S1C shows frequency of interpretations of immunohistochemistry staining patterns in the presence of TP53 gene mutations and the gene status in the presence of TP53‐positive stained samples (data from the studies that p53 status detected both by IHC and sequencing).
**Figure S2**. Funnel plot of all studies included in the present meta‐analysis.
**Figure S3**. Meta‐analysis of the pooled effect of p53 higher expression on survival stratified by histology and adjustment for standard prognostic variables.
**Figure S4**. Meta‐analysis of the pooled effect of p53 higher expression on survival only including studies performing IHC stratified by cut points and the forest plot of lower cut‐off value studies with pure ESCC cohorts.
**Figure S5**. Meta‐analysis of the pooled effect of p53 high expression on survival stratified by p53 expression analysis methodology, including all studies. *The p53 status detected both by IHC and sequencing in these studies.
**Figure S6**. Meta‐analysis of the pooled effect of p53 high expression on survival stratified by p53 expression analysis methodology, only including studies with pure ESCC cohorts. *The p53 status detected both by IHC and sequencing in these studies.
**Figure S7**. TP53 mutation rates detected in the studies, and size of the circle represents the sample size of the studies.

**Table S1**. Baseline characteristics of included studies
**Table S2**. Assessment of risk of bias
**Table S3**. Clinicopathological and survival data of all studies included in final meta‐analysis.
**Table S4**. Subgroup analyses
**Table S5**. Meta‐regression analysis of study factors associated interstudy heterogeneity
