# Supplementary material for: The high expression instead of mutation of p53 is predictive of overall survival in patients with esophageal squamous‐cell carcinoma: a meta‐analysis
Source: Cancer Med. 2016 Nov 23;6(1):54–66. doi: 10.1002/cam4.945 (PMC5269704; doi:10.1002/cam4.945)
Supplement: Supplementary file 1 — Figure S1. Analysis of ESCC patients in the IARC TP53 mutation database. Figure S1A depicts the corresponding IHC staining patterns of the type of TP53 gene mutations. Figure S1B shows how the TP53 mutation effect affects IHC staining patterns, where S1C shows frequency of interpretations of immunohistochemistry staining patterns in the presence of TP53 gene mutations and the gene status in the presence of TP53‐positive stained samples (data from the studies that p53 status detected both by IHC and sequencing). Figure S2. Funnel plot of all studies included in the present meta‐analysis. Figure S3. Meta‐analysis of the pooled effect of p53 higher expression on survival stratified by histology and adjustment for standard prognostic variables. Figure S4. Meta‐analysis of the pooled effect of p53 higher expression on survival only including studies performing IHC stratified by cut points and the forest plot of lower cut‐off value studies with pure ESCC cohorts. Figure S5. Meta‐analysis of the pooled effect of p53 high expression on survival stratified by p53 expression analysis methodology, including all studies. *The p53 status detected both by IHC and sequencing in these studies. Figure S6. Meta‐analysis of the pooled effect of p53 high expression on survival stratified by p53 expression analysis methodology, only including studies with pure ESCC cohorts. *The p53 status detected both by IHC and sequencing in these studies. Figure S7. TP53 mutation rates detected in the studies, and size of the circle represents the sample size of the studies. [file CAM4-6-54-s001.docx]

**The High Expression instead of Mutation of p53 is Predictive of Overall Survival in Patients with Esophageal Squamous-Cell Carcinoma: A Meta-Analysis**

## Supporting Information

**Summary Findings of ESCC in IARC TP53 Database Analysis and literatures**

The most current R17 version of the International Agency for Research on Cancer (IARC) TP53 database contains 5 studies that report on the prognostic effect of TP53 mutations in esophageal squamous cell carcinoma cohorts, all of which are found during our literature search.

All included 440 tumors in the database had information on their immunohistochemistry staining pattern. Positive immuno-staining occurred most commonly in G:C to A:T transitions and was mostly caused by Missense mutations (Supplementary Figure 1A and 1B). However, approximately 21% of *TP53* mutant tumors showed negative immuno-staining patterns (false negatives), as these are frequently frameshift mutations (Supplementary Figure 1C, data also from the studies that p53 status detected both by IHC and sequencing).

Therefore, studies performing p53 IHC analysis were assessed as being at low risk of biomarker measurement bias compared with studies performing gene sequencing or direct assessment of TP53 gene mutations analysis.

## Supplementary Figure Legends

Figure S1. Analysis of ESCC patients in the IARC TP53 mutation database. Supplementary Figure 1A depicts the corresponding IHC staining patterns of the type of TP53 gene mutations. Figure 1B shows how the TP53 mutation effect affects IHC staining patterns, where 1C shows frequency of interpretations of immunohistochemistry staining patterns in the presence of TP53 gene mutations and the gene status in the presence of TP53 positive stained samples (data from the studies that p53 status detected both by IHC and sequencing).

Figure S2. Funnel plot of all studies included in the present meta-analysis.

Figure S3. Meta-analysis of the pooled effect of p53 higher expression on survival stratified by histology and adjustment for standard prognostic variables.

Figure S4. Meta-analysis of the pooled effect of p53 higher expression on survival only including studies performing IHC stratified by cut points and the forest plot of lower cut-off value studies with pure ESCC cohorts.

Figure S5. Meta-analysis of the pooled effect of p53 high expression on survival stratified by p53 expression analysis methodology, including all studies. * The p53 status detected both by IHC and sequencing in these studies.

Figure S6. Meta-analysis of the pooled effect of p53 high expression on survival stratified by p53 expression analysis methodology, only including studies with pure ESCC cohorts. * The p53 status detected both by IHC and sequencing in these studies.

Figure S7. TP53 mutation rates detected in the studies, and size of the circle represents the sample size of the studies.

Figure S1


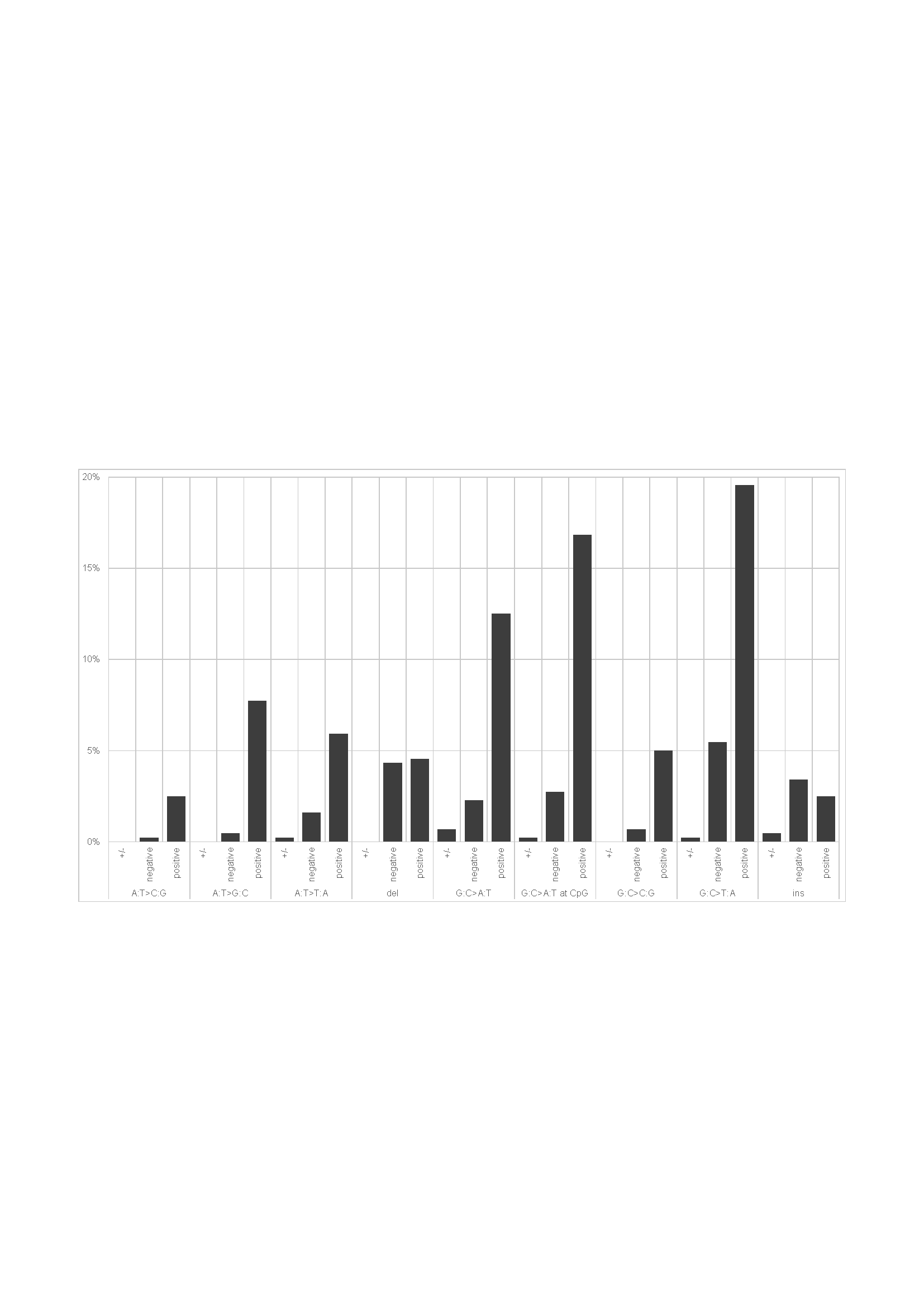


A. TP53 Mutation Types & Immunohistochemistry


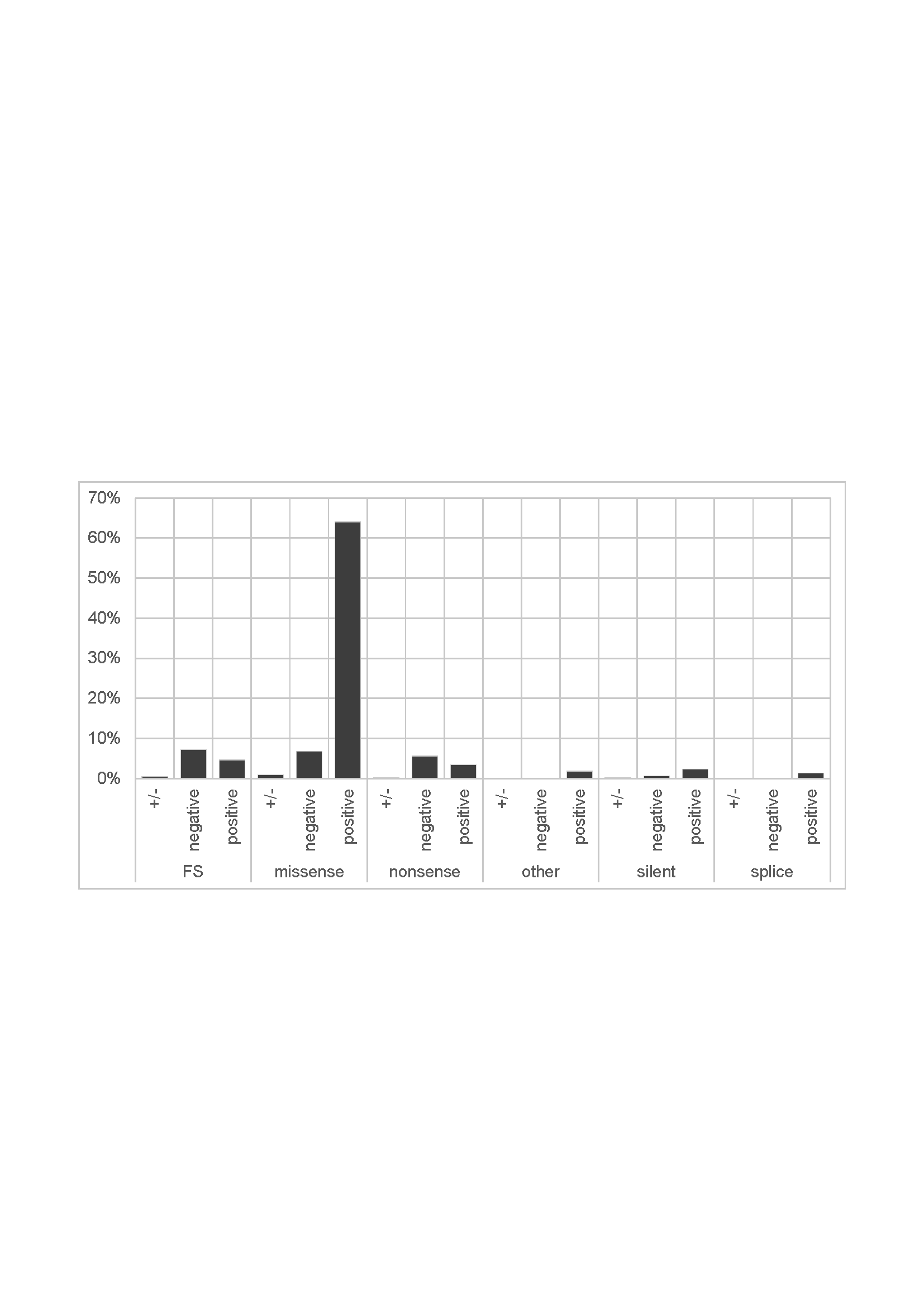


B. TP53 Mutation Effects & Immunohistochemistry


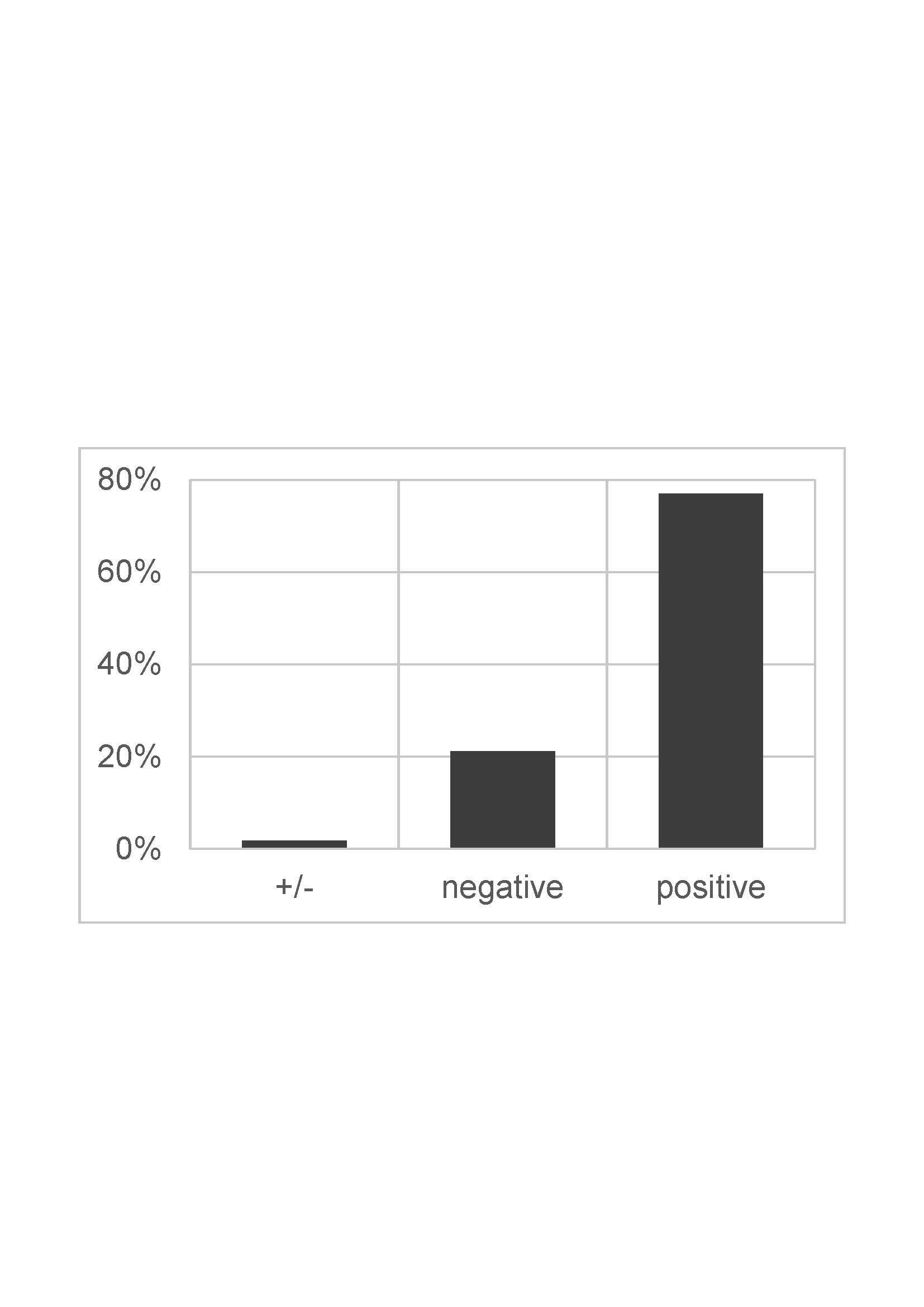

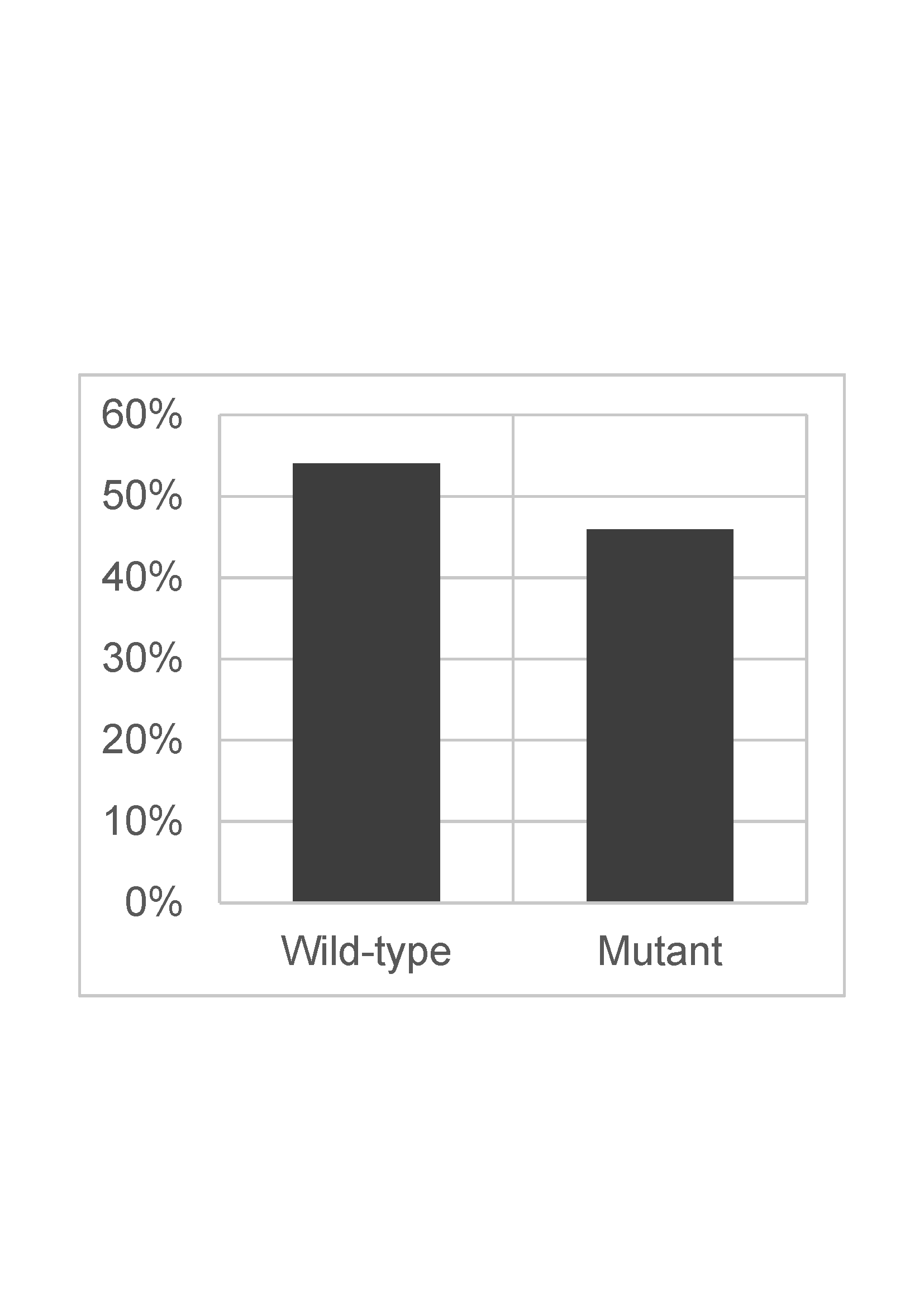


1. Staining Interpretation of Mutant Samples and *TP53* Status of the Positive Stained Samples

Figure S2


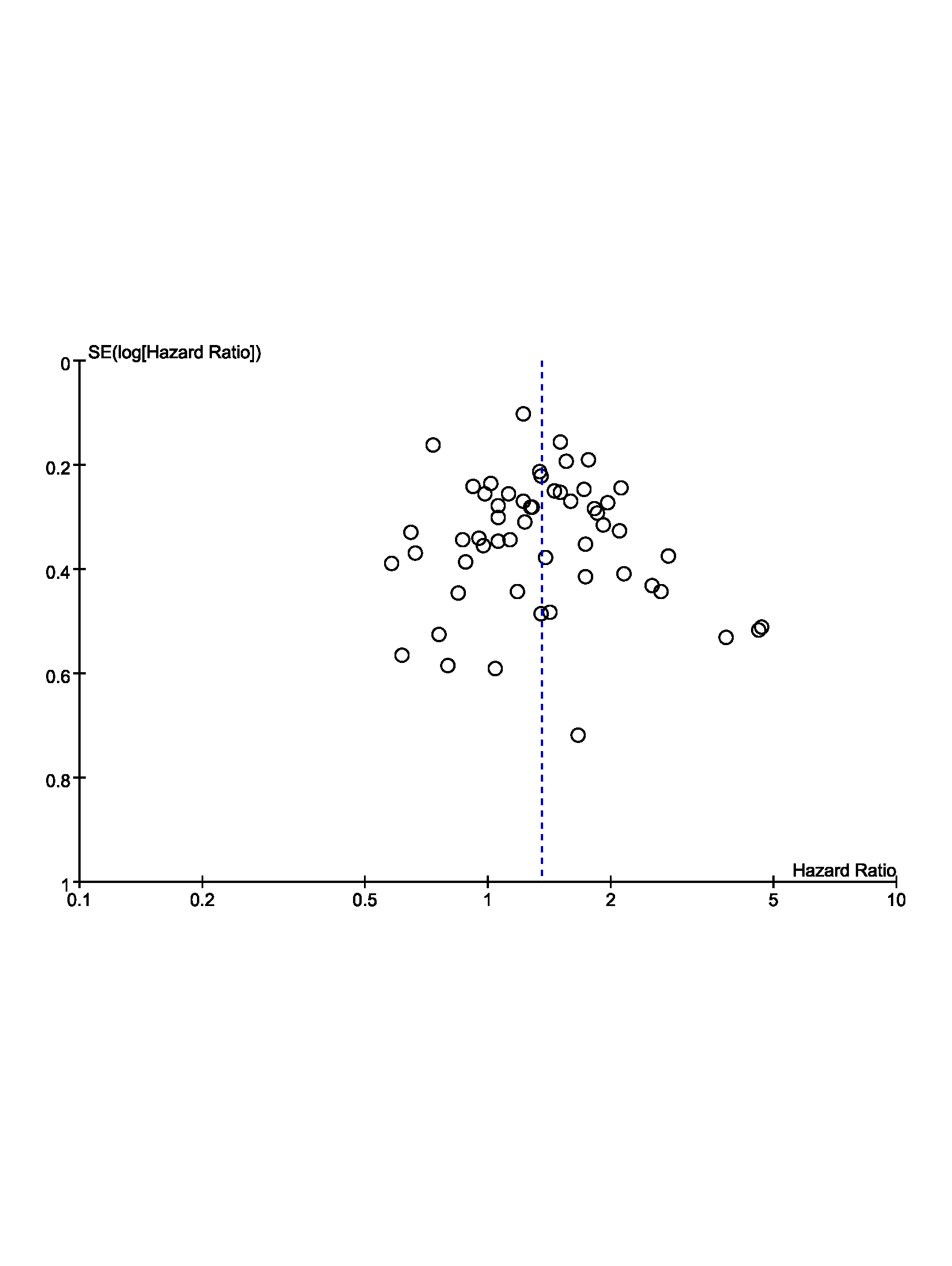


Figure S3.


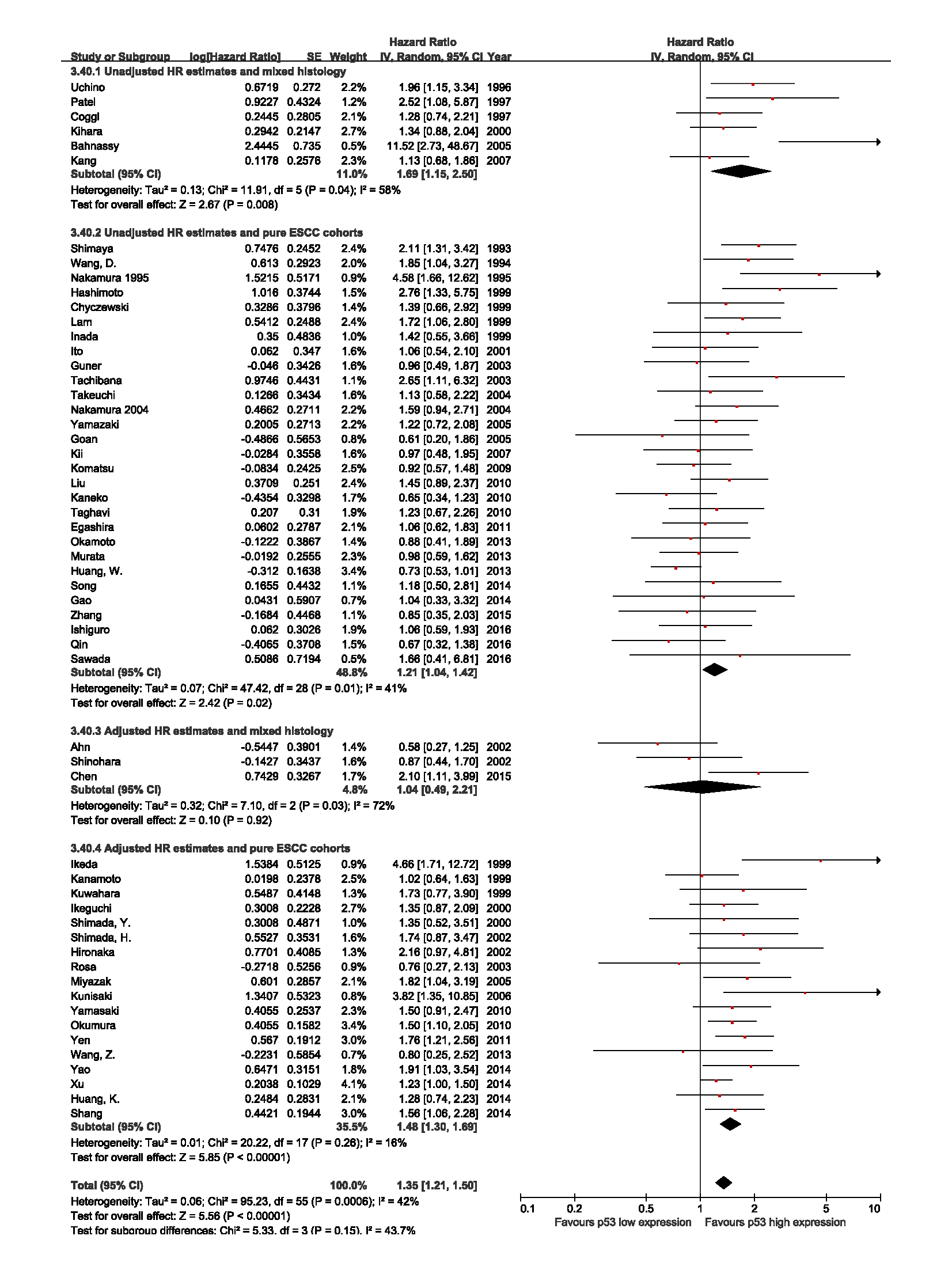


Figure S4.


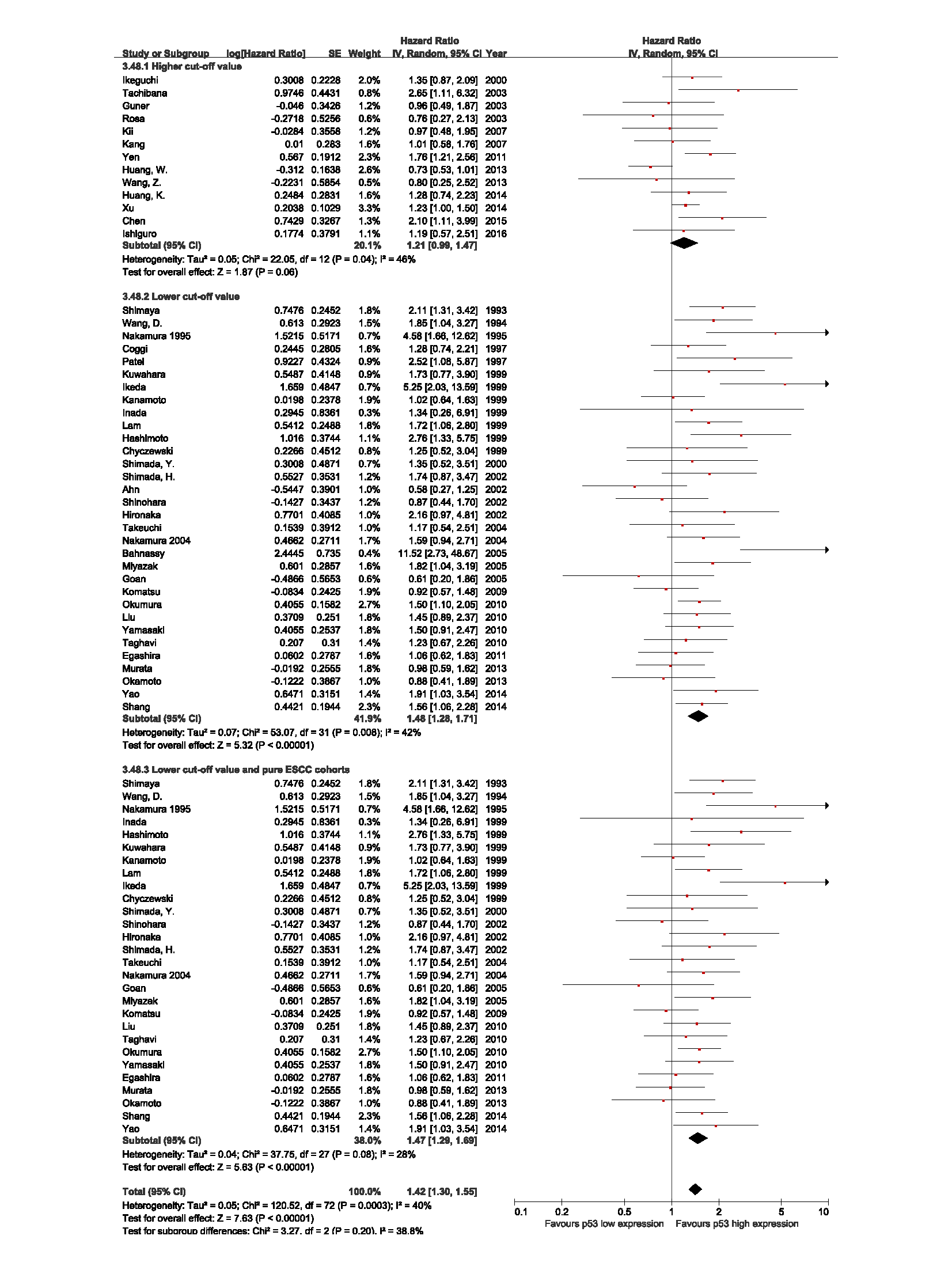


Figure S5.


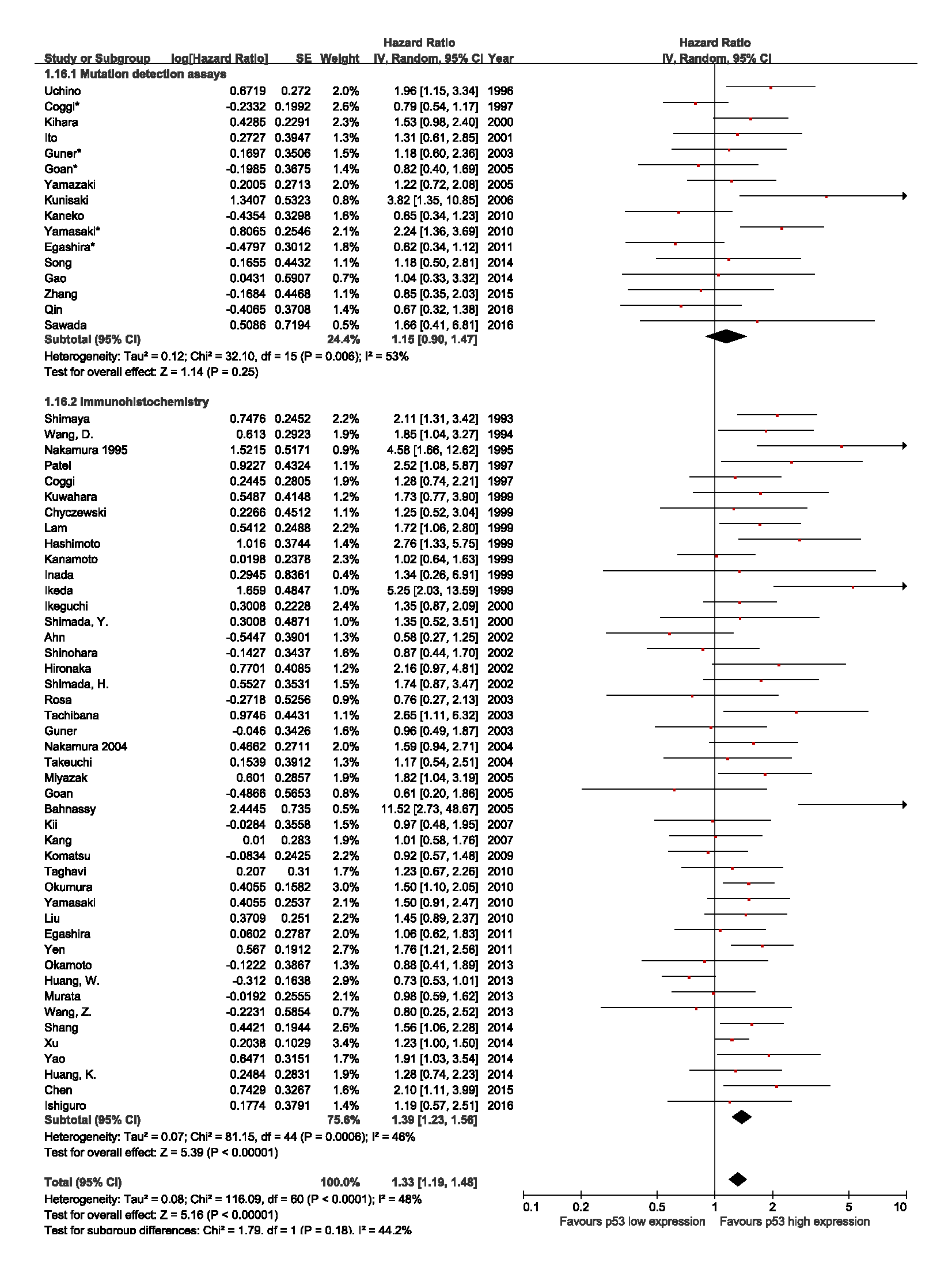


Figure S6.


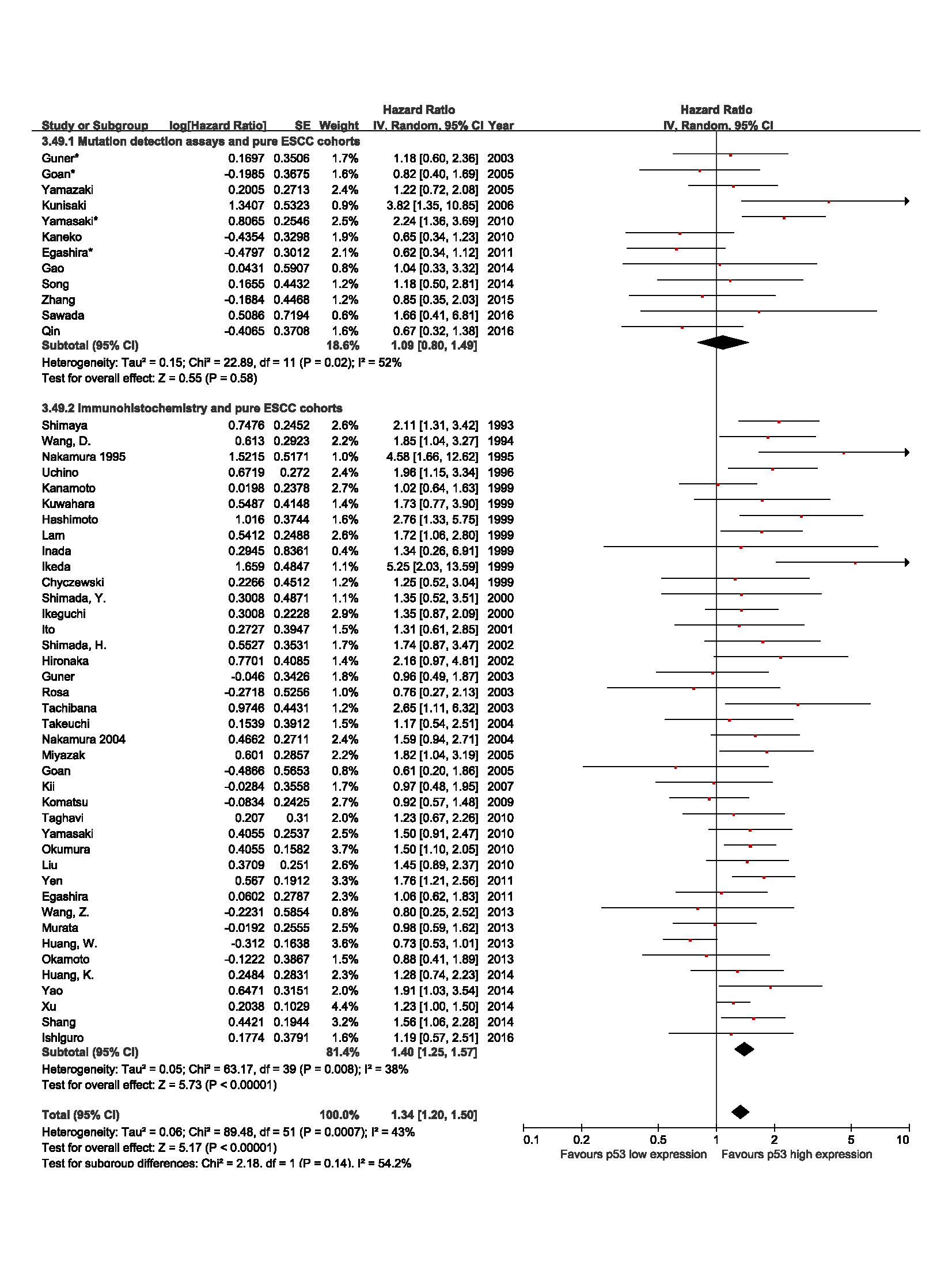


Figure S7.

Year

Tp53 mutation rates


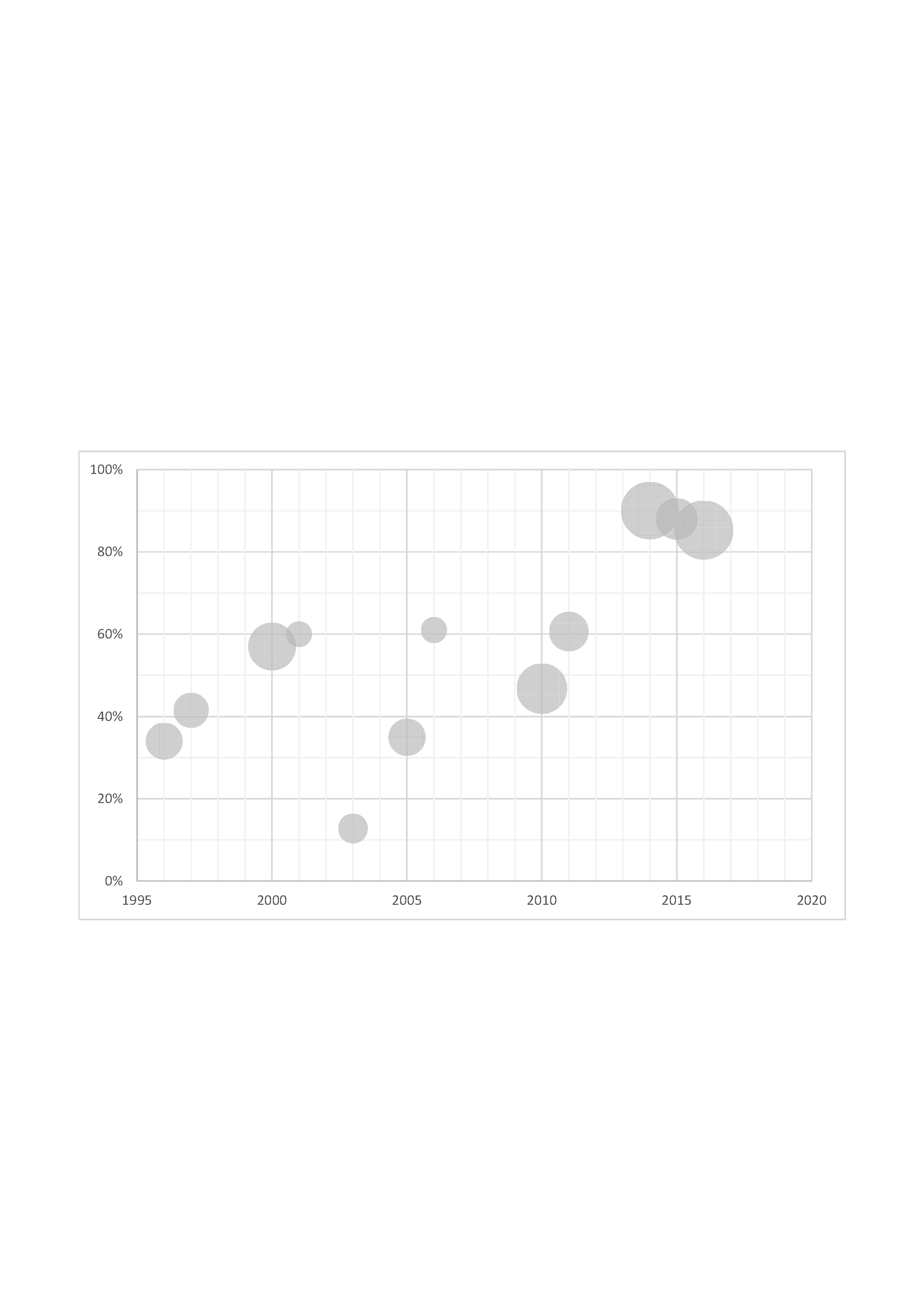


**References**[**1-56**](#_bookmark0)

1. Shimaya, K. *et al.* Significance of p53 expression as a prognostic factor in oesophageal squamous cell carcinoma. *Virchows Arch A Pathol Anat Histopathol*. **422**, 271-276 (1993).
2. Wang, D. Y. *et al.* High prevalence of p53 protein overexpression in patients with esophageal cancer in Linxian, China and its relationship to progression and prognosis. *Cancer*. **74**, 3089- 3096 (1994).
3. Nakamura, T. *et al.* Expression of p53 protein related to human papillomavirus and DNA ploidy in superficial esophageal carcinoma. *Surg Today*. **25**, 591-597 (1995).
4. Uchino, S. *et al.* Prognostic significance of the p53 mutation in esophageal cancer. *Jpn J Clin Oncol*. **26**, 287-292 (1996).
5. Coggi, G. *et al.* p53 protein accumulation and p53 gene mutation in esophageal carcinoma. A molecular and immunohistochemical study with clinicopathologic correlations. *Cancer*. **79**,

425-432 (1997).

1. Patel, D. D. *et al.* Clinical significance of p53, nm23, and bcl-2 in T3-4N1M0 oesophageal carcinoma: an immunohistochemical approach. *J Surg Oncol*. **65**, 111-116 (1997).
2. Chyczewski, L. *et al.* p53 protein expression in resected invasive esophageal cancer.

*Neoplasma*. **46**, 150-155 (1999).

1. Hashimoto, N., Tachibana, M., Dhar, D. K., Yoshimura, H. & Nagasue, N. Expression of p53 and RB proteins in squamous cell carcinoma of the esophagus: their relationship with clinicopathologic characteristics. *Ann Surg Oncol*. **6**, 489-494 (1999).
2. Ikeda, G., Isaji, S., Chandra, B., Watanabe, M. & Kawarada, Y. Prognostic significance of biologic factors in squamous cell carcinoma of the esophagus. *Cancer*. **86**, 1396-1405 (1999).
3. Inada, S., Koto, T., Futami, K., Arima, S. & Iwashita, A. Evaluation of malignancy and the prognosis of esophageal cancer based on an immunohistochemical study (p53, E-cadherin, epidermal growth factor receptor). *Surg Today*. **29**, 493-503 (1999).
4. Kanamoto, A. *et al.* No prognostic significance of p53 expression in esophageal squamous cell carcinoma. *J Surg Oncol*. **72**, 94-98 (1999).
5. Kuwahara, M. *et al.* p53, p21(Waf1/Cip1) and cyclin D1 protein expression and prognosis in esophageal cancer. *Dis Esophagus*. **12**, 116-119 (1999).
6. Lam, K. Y., Law, S., Tin, L., Tung, P. H. & Wong, J. The clinicopathological significance of p21 and p53 expression in esophageal squamous cell carcinoma: an analysis of 153 patients. *Am J Gastroenterol*. **94**, 2060-2068 (1999).
7. Ikeguchi, M. *et al.* Combined analysis of p53 and retinoblastoma protein expressions in esophageal cancer. *Ann Thorac Surg*. **70**, 913-917 (2000).
8. Kihara, C. *et al.* Mutations in zinc-binding domains of p53 as a prognostic marker of esophageal-cancer patients. *Jpn J Cancer Res*. **91**, 190-198 (2000).
9. Shimada, Y. *et al.* Histological response of cisplatin predicts patients' survival in oesophageal cancer and p53 protein accumulation in pretreatment biopsy is associated with cisplatin sensitivity. *Eur J Cancer*. **36**, 987-993 (2000).
10. Ito, T. *et al.* Prognostic value of p53 mutations in patients with locally advanced esophageal carcinoma treated with definitive chemoradiotherapy. *J Gastroenterol*. **36**, 303-311 (2001).
11. Ahn, M. J. *et al.* Clinical prognostic values of vascular endothelial growth factor, microvessel density,and p53 expression in esophageal carcinomas. *J Korean Med Sci*. **17**, 201-207 (2002).
12. Hironaka, S. *et al.* Biopsy specimen microvessel density is a useful prognostic marker in

patients with T(2-4)M(0) esophageal cancer treated with chemoradiotherapy. *Clin Cancer Res*.

**8**, 124-130 (2002).

1. Shimada, H. *et al.* Expression of angiogenic factors predicts response to chemoradiotherapy and prognosis of oesophageal squamous cell carcinoma. *Br J Cancer*. **86**, 552-557 (2002).
2. Shinohara, M. *et al.* Cell cycle-regulated factors in esophageal cancer. *Dis Esophagus*. **15**, 149-154 (2002).
3. Guner, D. *et al.* Multigene analysis of Rb pathway and apoptosis control in esophageal squamous cell carcinoma identifies patients with good prognosis. *Int J Cancer*. **103**, 445-454 (2003).
4. Rosa, A. R. *et al.* Prognostic value of p53 protein expression and vascular endothelial growth factor expression in resected squamous cell carcinoma of the esophagus. *Dis Esophagus*. **16**, 112-118 (2003).
5. Tachibana, M. *et al.* Postoperative chemotherapy vs chemoradiotherapy for thoracic esophageal cancer: a prospective randomized clinical trial. *Eur J Surg Oncol*. **29**, 580-587 (2003).
6. Nakamura, T. *et al.* Expression of p21(Waf1/Cip1) predicts response and survival of esophageal cancer patients treated by chemoradiotherapy. *Dis Esophagus*. **17**, 315-321 (2004).
7. Takeuchi, H. *et al.* Loss of p16INK4a expression is associated with vascular endothelial growth factor expression in squamous cell carcinoma of the esophagus. *Int J Cancer*. **109**, 483-490 (2004).
8. Bahnassy, A. A., Zekri, A. R., Abdallah, S., El-Shehaby, A. M. & Sherif, G. M. Human papillomavirus infection in Egyptian esophageal carcinoma: correlation with p53, p21, mdm2, C-erbB2 and impact on survival. *Pathol Int*. **55**, 53-62 (2005).
9. Goan, Y. G. *et al.* Deregulated p21(WAF1) overexpression impacts survival of surgically resected esophageal squamous cell carcinoma patients. *Ann Thorac Surg*. **80**, 1007-1016 (2005).
10. Miyazaki, T. *et al.* Predictors of response to chemo-radiotherapy and radiotherapy for esophageal squamous cell carcinoma. *Anticancer Res*. **25**, 2749-2755 (2005).
11. Yamazaki, K. *et al.* Increased E2F-1 expression via tumour cell proliferation and decreased apoptosis are correlated with adverse prognosis in patients with squamous cell carcinoma of the oesophagus. *J Clin Pathol*. **58**, 904-910 (2005).
12. Kunisaki, C. *et al.* Prognostic factors after chemoradiotherapy for patients with inoperable esophageal squamous cell carcinoma. *Hepatogastroenterology*. **53**, 366-371 (2006).
13. Kang, S. Y. *et al.* Low expression of Bax predicts poor prognosis in patients with locally advanced esophageal cancer treated with definitive chemoradiotherapy. *Clin Cancer Res*. **13**, 4146-4153 (2007).
14. Kii, T. *et al.* Evaluation of prognostic factors of esophageal squamous cell carcinoma (stage II-

III) after concurrent chemoradiotherapy using biopsy specimens. *Jpn J Clin Oncol*. **37**, 583- 589 (2007).

1. Komatsu, S. *et al.* Overexpression of SMYD2 relates to tumor cell proliferation and malignant outcome of esophageal squamous cell carcinoma. *Carcinogenesis*. **30**, 1139-1146 (2009).
2. Kaneko, K. *et al.* EGFR gene alterations as a prognostic biomarker in advanced esophageal squamous cell carcinoma. *Front Biosci (Landmark Ed)*. **15**, 65-72 (2010).
3. Liu, W. K., Jiang, X. Y., Zhang, M. P. & Zhang, Z. X. The relationship between HPV16 and

expression of cyclooxygenase-2, P53 and their prognostic roles in esophageal squamous cell carcinoma. *Eur J Gastroenterol Hepatol*. **22**, 67-74 (2010).

1. Okumura, H. *et al.* Nuclear expression of 14-3-3 sigma is related to prognosis in patients with esophageal squamous cell carcinoma. *Anticancer Res*. **30**, 5175-5179 (2010).
2. Taghavi, N. *et al.* Association of p53/p21 expression with cigarette smoking and prognosis in esophageal squamous cell carcinoma patients. *World J Gastroenterol*. **16**, 4958-4967 (2010).
3. Yamasaki, M. *et al.* p53 genotype predicts response to chemotherapy in patients with squamous cell carcinoma of the esophagus. *Ann Surg Oncol*. **17**, 634-642 (2010).
4. Egashira, A. *et al.* Loss of p53 in esophageal squamous cell carcinoma and the correlation with survival: analyses of gene mutations, protein expression, and loss of heterozygosity in Japanese patients. *J Surg Oncol*. **104**, 169-175 (2011).
5. Yen, C. C. *et al.* PML protein as a prognostic molecular marker for patients with esophageal squamous cell carcinomas receiving primary surgery. *J Surg Oncol*. **103**, 761-767 (2011).
6. Huang, W., Deng, B., Wang, R. W., Tan, Q. Y. & Jiang, Y. G. Expression of breast cancer anti- estrogen resistance 1 in relation to vascular endothelial growth factor, p53, and prognosis in esophageal squamous cell cancer. *Dis Esophagus*. **26**, 528-537 (2013).
7. Murata, A. *et al.* p53 immunohistochemical expression and patient prognosis in esophageal squamous cell carcinoma. *Med Oncol*. **30**, 728 (2013).
8. Okamoto, H. *et al.* Significance of CD133 expression in esophageal squamous cell carcinoma.

*World J Surg Oncol*. **11**, 51 (2013).

1. Wang, Z. B. *et al.* High p53 and MAP1 light chain 3A co-expression predicts poor prognosis in patients with esophageal squamous cell carcinoma. *Mol Med Rep*. **8**, 41-46 (2013).
2. Gao, Y. B. *et al.* Genetic landscape of esophageal squamous cell carcinoma. *Nat Genet*. **46**, 1097-1102 (2014).
3. Huang, K. *et al.* Elevated p53 expression levels correlate with tumor progression and poor prognosis in patients exhibiting esophageal squamous cell carcinoma. *Oncol Lett*. **8**, 1441- 1446 (2014).
4. Shang, L. *et al.* A panel of overexpressed proteins for prognosis in esophageal squamous cell carcinoma. *PLoS One*. **9**, e111045 (2014).
5. Song, Y. *et al.* Identification of genomic alterations in oesophageal squamous cell cancer.

*Nature*. **509**, 91-95 (2014).

1. Xu, X. L. *et al.* p53 is an independent prognostic factor in operable esophageal squamous cell carcinoma: a large-scale study with a long follow-up. *Med Oncol*. **31**, 257 (2014).
2. Yao, W. *et al.* Association of p53 expression with prognosis in patients with esophageal squamous cell carcinoma. *Int J Clin Exp Pathol*. **7**, 7158-7163 (2014).
3. Chen, J. *et al.* Analysis of the correlation between P53 and Cox-2 expression and prognosis in esophageal cancer. *Oncol Lett*. **10**, 2197-2203 (2015).
4. Zhang, L. *et al.* Genomic analyses reveal mutational signatures and frequently altered genes in esophageal squamous cell carcinoma. *Am J Hum Genet*. **96**, 597-611 (2015).
5. Ishiguro, H. *et al.* GADD45A expression is correlated with patient prognosis in esophageal cancer. *Oncol Lett*. **11**, 277-282 (2016).
6. Qin, H. D. *et al.* Genomic Characterization of Esophageal Squamous Cell Carcinoma Reveals Critical Genes Underlying Tumorigenesis and Poor Prognosis. *Am J Hum Genet*. **98**, 709-727 (2016).
7. Sawada, G. *et al.* Genomic Landscape of Esophageal Squamous Cell Carcinoma in a Japanese Population. *Gastroenterology*2016).
